# Beyond antigen dependence: Improving the therapeutic window of a signal 1 booster for adoptive cell therapy in solid tumors

**DOI:** 10.1016/j.omton.2026.201360

**Published:** 2026-10-28

**Authors:** Angelis M. Morales Rivera, Jiasen He, Candelaria Gomez-Manzano, Juan Fueyo, Hong Jiang

**Affiliations:** 1 Department of Neuro-Oncology, Department of Pediatrics, The University of Texas MD Anderson Cancer Center, Houston, TX, USA; 2 Department of Pediatrics, The University of Texas MD Anderson Cancer Center, Houston, TX, USA

## Main text

CAR T cell therapy has transformed the treatment of several hematologic malignancies, yet comparable success in solid tumors remains elusive because of heterogeneous antigen expression, restricted T cell trafficking, and an immunosuppressive tumor microenvironment (TME) that limits T cell activation, expansion, persistence, and effector function.^1^^,^^2^ Insufficient access to tumor antigen can further limit signal 1 and compromise engineered T cell fitness. Pharmacologically inducible MyD88/CD40 signaling modules have been developed to provide controlled activation and enhance CAR T cell activity.^3^^,^^4^ Although this strategy has improved efficacy in preclinical solid-tumor models, the phase 1 trial of the MyD88/CD40-equipped product BPX-601 CAR T cells was terminated following serious treatment-related toxicities.^5^ In the previous issue of *Molecular Therapy Oncology*, Radecki and colleagues addressed this challenge by engineering a MyD88/CD40 signal 1 booster (S1B) with an improved preclinical efficacy-safety profile, offering a potential path toward safer drug-controlled adoptive cell therapy (Figure 1).^6^

**Figure 1:**
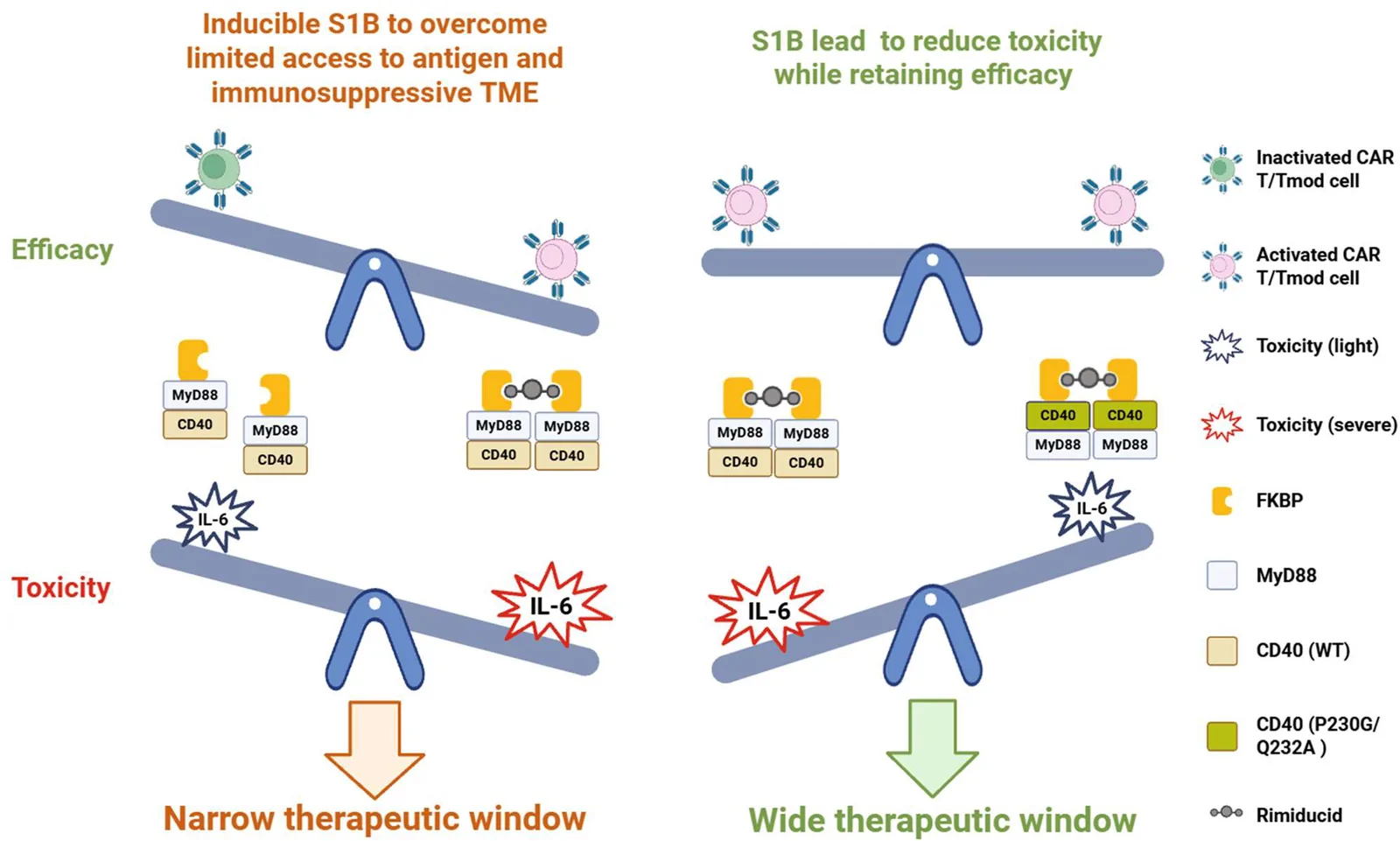
Modification of S1B to balance the efficacy and toxicity of engineered T cell therapy for solid tumors Rimiducid induces potent T cell activity by promoting dimerization of parental FKBP-MyD88/CD40 modules, but excessive activation can trigger inflammatory cytokine production, including IL-6, and associated toxicity. The S1B lead incorporates P230G/Q232A substitutions in CD40 to attenuate inflammatory cytokine production while preserving inducible T cell activity, thereby improving the efficacy-toxicity balance.

### Inducible MyD88/CD40 signaling: Powering T cells through a hostile *in vivo* environment

Once administered *in vivo*, CAR T cells embark on a journey through a hostile environment. They must first survive in circulation, where encounters with target antigen may be limited. Upon entering tumors, they face inhibitory checkpoints, suppressive immune populations and cytokines, metabolic stress, hypoxia, nutrient deprivation, and inadequate co-stimulation.^1^^,^^2^ Rather than eliminating these barriers, a MyD88/CD40 module activates NF-κB and associated survival and inflammatory programs, promoting T cell proliferation, persistence, cytokine production, and effector function.^3^^,^^4^ It may therefore enable engineered T cells to maintain their fitness and function despite, partially bypassing the functional consequences of, the hostile *in vivo* environment.

Foster et al. developed inducible MyD88/CD40 as a small-molecule-controlled module that enhanced CAR T cell proliferation and survival,^3^ whereas Mata et al. demonstrated enhanced proliferation, serial tumor cell killing, and antitumor activity in preclinical solid tumor models.^4^ In these earlier platforms, MyD88/CD40 primarily functioned as an inducible costimulatory signal cooperating with antigen-driven CAR or TCR signaling. Although LAT- and SLP76-based constructs generated acute signaling, they failed to sustain primary human T cell proliferation, whereas MyD88/CD40-based constructs supported prolonged expansion.^3^^,^^4^ These findings suggest that selected proximal TCR-signaling events may be insufficient to reproduce the durable consequences of antigen recognition, whereas broader survival and activation programs generated by MyD88/CD40 can support T cells during inadequate antigen stimulation.

A major translational advantage is pharmacological inducibility. Rimiducid induces dimerization of FKBP-fused MyD88/CD40 modules and downstream signaling. With a reported half-life of 4–12 h,^7^ rimiducid provides a means of temporally controlling this stimulation. Its administration can potentially be initiated, repeated, or withheld according to T cell expansion, tumor response, and toxicity, allowing activity to be dynamically managed during therapy rather than determined entirely before infusion.

### BPX-601: Clinical proof of concept reveals the therapeutic window

BPX-601 is a PSCA (prostate stem cell antigen)-targeted T cell product incorporating rimiducid-inducible MyD88/CD40 signaling.^5^ In a phase 1 trial in advanced pancreatic and prostate cancers, BPX-601 cells expanded, persisted, infiltrated tumors, and demonstrated antitumor activity. Among nine patients with metastatic castration-resistant prostate cancer (mCRPC), five achieved at least a 50% decrease in prostate-specific antigen, and partial radiographic responses were observed.^5^ The study also demonstrated re-dosability: mCRPC cohort 5B received a single rimiducid dose on day 7, whereas cohort 5C received weekly rimiducid beginning on day 7, allowing repeated pharmacological reactivation of previously infused BPX-601 cells.^5^

However, BPX-601 also exposed the liability of this powerful pathway. Rimiducid-induced activation increased inflammatory cytokines and was associated with cytokine release syndrome and other toxicities. Repeated activation in cohort 5C was associated with dose-limiting toxicities and treatment-related deaths, highlighting the need to preserve pharmacological control while attenuating inflammatory output.^5^ The study was terminated without establishment of a recommended phase 2 dose. Thus, BPX-601 demonstrated both sides of the therapeutic window: substantial biological and clinical activity but also the potential for excessive inflammatory signaling and serious toxicity.

### Engineering the signal 1 booster lead: Widening the therapeutic window

Rather than abandoning MyD88/CD40 because of its potency, Radecki et al. sought to tame the pathway. They screened variants to preserve rimiducid-dependent T cell expansion while reducing inflammatory cytokine production. The lead S1B contains P230G/Q232A substitutions within the CD40 TRAF1/2/3 recruitment region, attenuating this component of CD40 signaling while retaining drug-inducible T cells expansion.^6^^,^^8^ The lead produced substantially less IL-6 while maintaining activity in CAR T and Tmod cells (Figure 1).^6^ These findings suggest that proliferative and inflammatory outputs may be partially separable.

*In vivo*, the S1B lead retained rimiducid-dependent activity, including increased human T cell expansion and sustained effector activity, while reducing IL-6 production and avoiding the off-target toxicity observed with parental MyD88/CD40 in a surrogate normal-tissue model.^6^ These findings support the possibility of improving the efficacy-safety balance through selective attenuation of inflammatory signaling.

Furthermore, Tmod provides an additional layer of tumor selectivity by combining an activating CAR or TCR with an inhibitory receptor recognizing an HLA allele retained by normal tissues but lost by tumor cells through loss of heterozygosity. This logical NOT gate architecture permits killing of activating-antigen-positive, blocker-antigen-negative tumor cells while protecting normal cells that retain the blocker antigen.^9^ Incorporating S1B is intended to enhance T cell fitness without compromising this antigen-dependent safety logic.^6^

BPX-601 provides relevant clinical validation of drug-controlled MyD88/CD40 signaling, but CAR T/Tmod with S1B lead has not yet been evaluated clinically. Further studies must determine whether the improved preclinical efficacy-safety balance translates into a wider therapeutic window in patients. S1B lead should not be interpreted as eliminating the physical or immunological barriers of solid tumors. Rather, it may maintain CAR T/Tmod fitness during insufficient antigen exposure and reinforce activity within the immunosuppressive TME. It could also complement approaches that increase tumor inflammation or T cell recruitment, including radiotherapy, chemotherapy, cytokine or chemokine engineering, and oncolytic viruses.

### Toward a more effective and safer generation of drug-controlled cell therapies

The evolution of MyD88/CD40-based cell therapy is instructive. Earlier studies established its ability to enhance engineered T cell activity^3^^,^^4^; BPX-601 demonstrated clinical activity while exposing the consequences of excessive inflammatory signaling^5^; and Radecki and colleagues now take an important next step, engineering not only when MyD88/CD40 signaling occurs but also its strength and quality.^6^

If successfully translated to patients, the S1B lead may help convert the clinical promise of inducible MyD88/CD40 signaling into safer and potentially more effective adoptive cell therapies for solid tumors. Incorporation into Tmod may also support allogeneic, “off-the-shelf” approaches by combining enhanced functional fitness with HLA-based tumor selectivity. However, allogeneic compatibility, immune rejection, and clinical safety require independent evaluation before this broader potential can be realized.

## Declaration of interests

The authors declare no competing interests.

## Declaration of generative AI and AI-assisted technologies in the manuscript preparation process

During the preparation of this work the authors used ChatGPT in order to search for certain references and edit the language. After using this tool, the authors reviewed and edited the content as needed and take full responsibility for the content of the publication.
